# A Decision-Making Method with Grey Multi-Source Heterogeneous Data and Its Application in Green Supplier Selection

**DOI:** 10.3390/ijerph15030446

**Published:** 2018-03-03

**Authors:** Huifang Sun, Yaoguo Dang, Wenxin Mao

**Affiliations:** 1 College of Economics and Management, Nanjing University of Aeronautics and Astronautics, Nanjing 211106, China; iamdangyg@163.com; 2 School of Economics and Management, Southeast University, Nanjing 211189, China; maowenxin@seu.edu.cn

**Keywords:** grey multi-source heterogeneous data, kernel and greyness degree, multi-attribute decision making, green supplier selection

## Abstract

In view of the multi-attribute decision-making problem that the attribute values are grey multi-source heterogeneous data, a decision-making method based on kernel and greyness degree is proposed. The definitions of kernel and greyness degree of an extended grey number in a grey multi-source heterogeneous data sequence are given. On this basis, we construct the kernel vector and greyness degree vector of the sequence to whiten the multi-source heterogeneous information, then a grey relational bi-directional projection ranking method is presented. Considering the multi-attribute multi-level decision structure and the causalities between attributes in decision-making problem, the HG-DEMATEL method is proposed to determine the hierarchical attribute weights. A green supplier selection example is provided to demonstrate the rationality and validity of the proposed method.

## 1. Introduction

Multi-attribute decision-making is a problem in which a decision-maker evaluates a finite set of alternatives associated with multiple attributes. It is widely used in various areas such as management [1,2], the environment [3], economy [4,5], technology [6] and engineering [7,8]. Due to the uncertainty of the actual decision-making environment and the limitations of human cognition, the decision-making information usually consists of grey numbers for a decision maker in the evaluation of the alternatives, such as interval grey numbers [9,10] and extended grey numbers [11]. With the aggravation of the environmental complexity, the decision-making problem also requires multi-source decision information to evaluate the potential alternatives objectively and comprehensively. However, the diversity of information sources often forms a heterogeneous data sequence that contains different types of data. In this case, the traditional single type of grey information can no longer meet the requirements of the actual modeling. Therefore, grey multi-source heterogeneous data are presented, which can deal with higher complexity and uncertainty to describe the potential alternative’s comprehensive performance more accurately.

Nowadays, the current research about the multi-attribute decision-making method has resulted in many significant achievements, such as AHP [12], TODIM [13], ELECTRE III [14], TOPSIS [15] and VIKOR [16]. Grey relational analysis (GRA) is an effective method to solve multi-attribute decision-making problem in grey system theory [17]. Its basic idea is to judge whether or not different data sequences are closely associated according to the geometric shapes of their sequence curves. The more similar the curves are, the greater the grey relational degree is, and vice versa. The GRA method has the advantages that it is unnecessary to take the sample size and typical distribution regularity into consideration. At present, this method has been widely used in practice. Kolhapure et al. [18] used the GRA method to study the problem of geometrical optimization of strain gauge force transducers. Kirubakaran and Ilangkumaran [19] developed a hybrid MADM model which combined FAHP, GRA and TOPSIS to select an optimum maintenance strategy. Li and Zhao [20] integrated GRA and VIKOR for evaluating performance of industrial plants. Liu et al. [21] proposed a dynamic fuzzy GRA method to select emergency treatment technology. In addition, many scholars also improved the GRA method theoretically, including the optimization of several GRA models [22], the construction of grey relational degree [23] and the weights determination in GRA [24]. Throughout the relevant literature of GRA, whether it is related to application or to methodological research, the employed attribute values usually form a single type of decision-making information. But in the case of GRA whose attribute values are a mixture of different data types of grey information from multiple sources, the study is still relatively scarce. Therefore, we extend the GRA method to accommodate grey multi-source heterogeneous data.

Additionally, in practical decision-making, the decision problem usually presents a multi-attribute multi-level decision-making structure, and the evaluation attributes are not entirely independent, but mostly causal. Most of the existing decision-making methods assume that the attributes are independent of each other, and the causalities between attributes are neglected. Decision Making Trial and Evaluation Laboratory (DEMATEL) is a method for analyzing complex system factors. It can recognize the causalities between complex social factors and identify the key elements based on graph theory [25]. In the DEMATEL method, the attributes are divided into a set of cause attributes and a set of effect attributes, wherein the cause attributes have a certain influence on the effect attributes. According to the influencing relationships, the relative importance between attributes can be determined. It can be seen that the DEMATEL method can effectively handle the causalities between attributes and determine the attribute weights. At present, the DEMATEL method has been widely applied in the fields of green supply chain management [26], sustainable supply chain management [27], sustainable consumption and production [28], risk analysis [29] and other fields. The determination of the initial direct relation matrix usually requires group multi-expert information aggregation in the DEMATEL method. However, the current papers mostly use the mean method to integrate the initial direct relation matrix given by each expert, and the linear environment hypothesis used by the mean method is ignored. In view of the above problems, this paper endeavors to make attempts from the following aspects:

- The fusion of different data types of grey information from multiple sources is processed by kernel and greyness degree which are the common information characteristics of grey multi-source heterogeneous data.
- In order to measure the proximity of a selected alternative to the ideal solution and the negative ideal solution comprehensively and accurately, a grey relational bi-directional projection method is proposed based on kernel and greyness degree.
- The Hierarchical Group-Decision Making Trial and Evaluation Laboratory (HG-DEMATEL) method is proposed based on analyzing the causalities between attributes. It can effectively use group decision-making information and determine the hierarchical attribute weights.
- The validity of the proposed method is verified in the practical decision problem of green supplier selection.

The remainder of this paper is organized as follows: in Section 2 the literature review on green supplier selection is presented. In Section 3 the related definitions for this paper are introduced. In Section 4 the decision-making method is proposed based on grey multi-source heterogeneous data. In Section 5 the proposed method is applied to evaluate the potential green suppliers. In Section 6 we perform the result analyses, which include the causalities analysis, the sensitivity analysis and the comparative analysis. Finally, some conclusions and future research directions are presented in Section 7.

## 2. Literature Review

Green supplier selection is actually a kind of multiple attribute decision-making (MADM) problem. Recently, a variety of MADM methods for green supplier selection have been proposed. Govindan et al. [30] utilized AHP method to identify the essential barriers for green supply chain management. Yu and Hou [31] proposed a modified MMAHP method for green supplier selection and applied this method in the area of automobile manufacturing. In the above methods, they are needed to keep on detecting and adjusting the consistency and the deviation of the comparison matrix. Lin et al. [32] applied ANP method to solve the green supplier selection problem for an electronic manufacturing company. The method was shown to produce reliable and stable results for MADM problems with incomplete information. Govindan et al. [33] developed a novel PROMETHEE method that was similar to AHP and ANP to rank the green suppliers in food supply chain. Roshandel et al. [34] proposed a hierarchical TOPSIS method to evaluate and select the potential suppliers in supply chain management.

Besides that, the optimization models have also been widely adopted in the field of green supplier selection. Azadi et al. [35] proposed an extended data envelopment analysis (DEA) method to measure the effectiveness, efficiency and productivity of the potential suppliers in uncertain environment. Teresa and Jennifer [36] utilized an augmented DEA method to evaluate the suppliers, and the experiments showed that the proposed augmented DEA model was superior to the basic DEA model as well as the cross-efficiency and super-efficiency models. Ghayebloo et al. [37] put forward a bi-objective model for green supplier selection and disassembly of products. Meanwhile, the mixed integer programming model is proposed to solve a forward/reverse logistic network problem. Amin and Zhang [38] developed a multi-objective linear programming model to make decisions on the supplier selection and refurbishing site determination. The proposed model can take the supplier selection, order allocation, and CLSC network configuration into consideration synchronously. The above studies mostly used a single method to solve the green supplier selection problem. However, many complex decision-making problems often cannot be solved by a single method.

In order to deal with the green supplier selection problem with the higher complexity, many scholars presented the integrated hybrid methods. Stanujkic et al. [39] studied the ELECTRE and PROMETHEE, which were devoted to compare the potential alternatives’ characteristics and performances. Qin et al. [40] developed an extended TODIM method based on prospect theory to solve the green supplier selection problem. Tsui and Wen [41] put forward a hybrid MAGDM method based on AHP, entropy and ELECTRE III for green supplier selection, which considered the environmental issue. Hamdan and Cheaitou [42] integrated an MADM and multi-objective optimization method for green supplier selection. Bai et al. [43] proposed a hybrid method based on rough set theoretic and clustering means methods. The proposed method can well solve the complex investment decision problems in the area of green supplier development and business supplier development practices. Luthra et al. [44] integrated AHP, VIKOR, a multi-criteria optimization and compromise solution approach for evaluating supplier selection.

According to the review and analysis on green supplier selection based on the above-mentioned references, we can see that most of the above decision-making methods assume that the attributes are independent of each other, and the causal relationships between attributes are neglected. Hence, it is necessary to study the decision problem with causalities between attributes. Meanwhile, the single uncertain decision-making information has been widely adopted in green supplier selection problems. However, little attention has been paid to the different types of uncertain mixed grey information from multiple sources to dispose multi-attribute green supplier selection. Therefore, it is advantageous to study the MADM method and its preference information under the grey multi-source heterogeneous information. This does not only improve the model ability of higher complexity and uncertainty, but also handle the green supplier selection problems with uncertain decision information.

## 3. Preliminaries

In real life, decision-making information is usually uncertain due to the difficulty of information acquisition and the limitation of decision maker’s own cognition. In most cases, a number is called a grey number ⊗, whose exact value is unknown but a range within that the value lies is known. The grey number ⊗ with upper bound $\bar{a}$ and lower bound $\underline{a}$ is called an interval grey number, denoted as $⊗\in \left[\underline{a},\bar{a}\right]$, where $\underline{a}<\bar{a}$.

**Definition** **1** [45]**.***Assume that the background or universe of an interval grey number* $⊗\in \left[\underline{a},\bar{a}\right]$, $\left(\underline{a}<\bar{a}\right)$ *is* Ω*, and let* μ(⊗) *be the measurement on the number field of interval grey number* ⊗*, then* g°(⊗)=μ(⊗)/μ(Ω) *is called the greyness degree of the interval grey number* ⊗*. In the absence of value distribution information of interval grey number* ⊗*,* $\hat{⊗}=\frac{\underline{a}+\bar{a}}{2}$ *is referred to as the kernel of interval grey number ⊗.*

Particularly, when $\underline{a}=\bar{a}=a$, $⊗\in \left[\underline{a},\bar{a}\right]$ will be degenerated to a real number a. The kernel and greyness degree of a real number are its own value and 0, respectively. Interval grey number is usually represented by a closed interval, but sometimes the continuous interval cannot completely reveal the uncertainty of given information. In view of this, Yang [46] proposed the definition of extended grey number.

**Definition** **2** [46]**.** *If* ${⊗}^{\pm }$ *is a union set of a set of interval sets, then* ${⊗}^{\pm }$ *is called extended grey number, denoted as* ${⊗}^{\pm }=\overset{n}{\underset{i=1}{\cup }}\left[{\underline{a}}_{i},{\bar{a}}_{i}\right]$*, where* 0<n<∞*,* $\left[{\underline{a}}_{i},{\bar{a}}_{i}\right]\cap \left[{\underline{a}}_{j},{\bar{a}}_{j}\right]=Ø$ (i≠j)*,* ${\bar{a}}_{i-1}\leq {\underline{a}}_{i}\leq {\bar{a}}_{i}\leq {\underline{a}}_{i+1}$ ${⊗}^{-}=inf_{{\underline{a}}_{i}\in {⊗}^{\pm }}{\underline{a}}_{i}$ *and* ${⊗}^{+}=sup_{{\bar{a}}_{i}\in {⊗}^{\pm }}{\bar{a}}_{i}$ *are called the lower and upper bounds of ${⊗}^{\pm }$ respectively.*

On the basis of the relevant definitions of the kernel and greyness degree of an interval grey number [45], the definitions of the kernel and greyness degree of an extended grey number are given as follows.

**Definition** **3.** *Assume that the kernel of extended grey number* ${⊗}^{\pm }=\overset{n}{\underset{i=1}{\cup }}\left[{\underline{a}}_{i},{\bar{a}}_{i}\right]$ *is* ${\hat{⊗}}^{\pm }$*, then*

*(1)*  If the probability distribution of ${⊗}^{\pm }$ *is unknown, then ${\hat{⊗}}^{\pm }=\frac{1}{n}\sum_{i=1}^{n}{\hat{a}}_{i}$;*
*(2)*  If the probability distribution of ${⊗}^{\pm }$ *is known, then* ${\hat{⊗}}^{\pm }=\sum_{i=1}^{n}p_{i}{\hat{a}}_{i}$*, where* $p_{i}$ *and* ${\hat{a}}_{i}$ *are the probability and the kernel of interval grey number* ${⊗}_{i}\in \left[{\underline{a}}_{i},{\bar{a}}_{i}\right]$ *respectively, and the following conditions hold:* $p_{i}>0$*, $\sum_{i=1}^{n}p_{i}=1$.*

**Definition** **4.** *Assume that the background or universe of extended grey number* ${⊗}^{\pm }=\overset{n}{\underset{i=1}{\cup }}\left[{\underline{a}}_{i},{\bar{a}}_{i}\right]$ *is* Ω*,* $\mu ({⊗}_{i})$ *is the measurement on number field of interval grey number* ${⊗}_{i}\in \left[{\underline{a}}_{i},{\bar{a}}_{i}\right]$*, then greyness degree* $g^{\circ }({⊗}^{\pm })$ *of extended grey number* ${⊗}^{\pm }$ *is defined as follows:*

*(1)*  If the probability distribution of ${⊗}^{\pm }$ *is unknown, then $g^{\circ }({⊗}^{\pm })=\sum_{i=1}^{n}\mu ({⊗}_{i})/\mu (\Omega )$;*
*(2)*  If the probability distribution of ${⊗}^{\pm }$ *is known, then* $g^{\circ }({⊗}^{\pm })=\sum_{i=1}^{n}p_{i}\mu ({⊗}_{i})/\mu (\Omega )$*, where* $p_{i}$ *is the probability of interval grey number* ${⊗}_{i}\in \left[{\underline{a}}_{i},{\bar{a}}_{i}\right]$*, and conditions hold:* $p_{i}>0$*, $\sum_{i=1}^{n}p_{i}=1$.*

**Definition** **5.** *If the decision-making information obtained from information sources 1, 2,* ⋯*,* m *is a data set which is a mixture of interval grey numbers, extended grey numbers and real numbers. (Note: a real number is a special grey number whose greyness degree is 0), then the sequence composed of the mixed data from the data set is called grey multi-source heterogeneous data sequence.*

## 4. The Proposed Decision-Making Method

### 4.1. Problem Description

In a multi-attribute decision-making problem, $S=\left\{S_{1},S_{2},\cdots ,S_{m}\right\}$ is a set of alternatives, and $B=\left\{b_{1},b_{2},\cdots ,b_{k},b_{k+1},b_{k+2},\cdots ,b_{s},b_{s+1},b_{s+2},\cdots ,b_{n}\right\}$ is a set of attributes. The weight vector of the attributes is $W=(w_{1},w_{2},\cdots ,w_{n})$, where $w_{j}\in [0,1]$, $\sum_{j=1}^{n}w_{j}=1$. Let the attribute value of the alternative $S_{i}(i=1,2,\cdots ,m)$ about the attribute $b_{j}(j=1,2,\cdots ,k)$ be denoted as $a_{ij}$, which takes the form of real number; the attribute value of the alternative $S_{i}$ about the attribute $b_{j}(j=k+1,k+2,\cdots ,s)$ is denoted as $b_{ij}(⊗)\in \left[{\underline{b}}_{ij},{\bar{b}}_{ij}\right]$, which takes the form of interval grey number; the attribute value of the alternative $S_{i}$ about the attribute $b_{j}(j=s+1,s+2,\cdots ,n)$ is denoted as $c_{ij}({⊗}^{\pm })=\overset{h}{\underset{l=1}{\cup }}\left[{\underline{c}}_{ij}^{l},{\bar{c}}_{ij}^{l}\right]$ $\left(\left[{\underline{c}}_{ij}^{l},{\bar{c}}_{ij}^{l}\right]\cap \left[{\underline{c}}_{ij}^{v},{\bar{c}}_{ij}^{v}\right]=Ø,l\neq v\right)$, which takes the form of extended grey number. Then the comprehensive decision matrix $H=(a\;b(⊗)\;c({⊗}^{\pm }))$ is obtained by putting $a={\left(a_{ij}\right)}_{m\times k}$, $b(⊗)={\left(b_{ij}(⊗)\right)}_{m\times (s-k)}$, $c({⊗}^{\pm })={\left(c_{ij}({⊗}^{\pm })\right)}_{m\times (n-s)}$.

To eliminate the dimensions of the different attributes and to increase comparability, the decision matrix $H=(a\;b(⊗)\;c({⊗}^{\pm }))$ needs to be normalized. The normalized decision matrix $Q=(y\;d(⊗)\;x({⊗}^{\pm }))$, and the formulas for standardizing $H=(a\;b(⊗)\;c({⊗}^{\pm }))$ are given as follows.

For a real number, let $a_{j}^{-}=\underset{i}{\min}(a_{ij})$, $a_{j}^{+}=\underset{i}{\max}(a_{ij})$, i=1,2,⋯,m, j=1,2,⋯,k, then $\mu_{j}=a_{j}^{+}-a_{j}^{-}$ is called the range or measurement of universe of attribute $b_{j}$.

For the benefit attribute $b_{j}$, we write:

(1) $$y_{ij}^{*}=\frac{a_{ij}-a_{j}^{-}}{\mu_{j}}$$

For the cost attribute $b_{j}$, we write:

(2) $$y_{ij}^{\nabla }=\frac{a_{j}^{+}-a_{ij}}{\mu_{j}}$$

For interval grey number, let $b_{j}^{-}=\underset{i}{\min}({\underline{b}}_{ij})$, $b_{j}^{+}=\underset{i}{\max}({\bar{b}}_{ij})$, i=1,2,⋯,m, j=k+1,k+2,⋯,s, then $\mu_{j}=b_{j}^{+}-b_{j}^{-}$ is called the range or measurement of universe of attribute $b_{j}$.

For the benefit attribute $b_{j}$, we write:

(3) $${\underline{d}}_{ij}^{*}=\frac{{\underline{b}}_{ij}-b_{j}^{-}}{\mu_{j}},\;{\bar{d}}_{ij}^{*}=\frac{{\bar{b}}_{ij}-b_{j}^{-}}{\mu_{j}}$$

For the cost attribute $b_{j}$, we write:

(4) $${\underline{d}}_{ij}^{\nabla }=\frac{b_{j}^{+}-{\bar{b}}_{ij}}{\mu_{j}},\;{\bar{d}}_{ij}^{\nabla }=\frac{b_{j}^{+}-{\underline{b}}_{ij}}{\mu_{j}}$$

For extended grey number, let $c_{j}^{-}=\underset{i}{\min}({\underline{c}}_{ij}^{1})$, $c_{j}^{+}=\underset{i}{\max}({\bar{c}}_{ij}^{h})$, i=1,2,⋯,m, j=s+1,s+2,⋯,n, then $\mu_{j}=c_{j}^{+}-c_{j}^{-}$ is called the range or measurement of universe of attribute $b_{j}$.

For the benefit attribute $b_{j}$, we write:

(5) $${\underline{x}}_{ij}^{l*}=\frac{{\underline{c}}_{ij}^{l}-c_{j}^{-}}{\mu_{j}},\;{\bar{x}}_{ij}^{l*}=\frac{{\bar{c}}_{ij}^{l}-c_{j}^{-}}{\mu_{j}}$$

For the cost attribute $b_{j}$, we write:

(6) $${\underline{x}}_{ij}^{l\nabla }=\frac{c_{j}^{+}-{\bar{c}}_{ij}^{l}}{\mu_{j}},\;{\bar{x}}_{ij}^{l\nabla }=\frac{c_{j}^{+}-{\underline{c}}_{ij}^{l}}{\mu_{j}}$$

### 4.2. Grey Relational Bi-Directional Projection Ranking Method Based on Kernel and Greyness Degree

Although grey multi-source heterogeneous data have different data structures and grey information characteristics, it belongs to the category of “grey numbers”. Therefore, it can be aggregated by using the kernel and greyness degree which are the common information characteristics of grey multi-source heterogeneous data.

In decision-making process, it is generally required that the optimal alternative should be as close as possible to the ideal solution and as far away as possible from the negative ideal solution. Based on this, in order to measure the proximity of a selected alternative to the ideal solution and the negative ideal solution comprehensively and accurately, the grey relational bi-directional projection method based on kernel and greyness degree is proposed to rank the alternatives. The proposed method integrates grey relational analysis theory with vector projection principle, and considers the effect of entire attribute space. The single direction deviation can be avoided especially when the sample size of attribute is spare and data is discretized.

**Definition** **6.** *Assume that a vector constituted by a grey multi-source heterogeneous data sequence is* $J_{i}(\overset{↔}{⊗})=(y_{i1},y_{i2},\cdots ,y_{ik},d_{i(k+1)}(⊗),d_{i(k+2)}(⊗),\cdots ,d_{is}(⊗),x_{i(s+1)}({⊗}^{\pm }),x_{i(s+2)}({⊗}^{\pm }),\cdots ,x_{in}({⊗}^{\pm }))$*, then the vector* ${\hat{\bar{⊗}}}_{i}$ *composed of “the whole kernel” in* $Y_{i}(\overset{↔}{⊗})$ *is called kernel vector of* $Y_{i}(\overset{↔}{⊗})$*, and the vector* ${\bar{g}}_{i}^{\circ }$ *composed of “the whole greyness degrees” in* $Y_{i}(\overset{↔}{⊗})$ *is called the greyness degree vector of* $Y_{i}(\overset{↔}{⊗})$*, namely*

(7) $${\hat{\bar{⊗}}}_{i}=(y_{i1},y_{i2},\cdots ,y_{ik},{\hat{⊗}}_{i(k+1)},{\hat{⊗}}_{i(k+2)},\cdots ,{\hat{⊗}}_{is},{\hat{⊗}}_{i(s+1)}^{\pm },{\hat{⊗}}_{i(s+2)}^{\pm },\cdots ,{\hat{⊗}}_{in}^{\pm })$$

(8) $${\bar{g}}_{i}^{\circ }=(0_{i1},0_{i2},\cdots ,0_{ik},g_{i(k+1)}^{{}^{\circ}}(⊗),g_{i(k+2)}^{{}^{\circ}}(⊗),\cdots ,g_{is}^{{}^{\circ}}(⊗),g_{i(s+1)}^{{}^{\circ}}({⊗}^{\pm }),g_{i(s+2)}^{{}^{\circ}}({⊗}^{\pm }),\cdots ,g_{in}^{{}^{\circ}}({⊗}^{\pm }))$$

*where* $y_{ij}$ (j=1,2,⋯,k) *is a real number,* $d_{ij}(⊗)$ (j=k+1,k+2,⋯,s) *is an interval grey number, and* $x_{ij}({⊗}^{\pm })$ (j=s+1,s+2,⋯,n) *is an extended grey number.*

**Definition** **7.** *Let* ${\hat{\bar{⊗}}}^{+}=({\hat{\bar{⊗}}}_{1}^{+},{\hat{\bar{⊗}}}_{2}^{+},\cdots ,{\hat{\bar{⊗}}}_{n}^{+})$ *and* ${\hat{\bar{⊗}}}^{-}=({\hat{\bar{⊗}}}_{1}^{-},{\hat{\bar{⊗}}}_{2}^{-},\cdots ,{\hat{\bar{⊗}}}_{n}^{-})$ *be the positive and negative ideal kernel vector respectively, and let* ${\bar{g}}^{\circ +}=({\bar{g}}_{1}^{\circ +},{\bar{g}}_{2}^{\circ +},\cdots ,{\bar{g}}_{n}^{\circ +})$ *and* ${\bar{g}}^{\circ -}=({\bar{g}}_{1}^{\circ -},{\bar{g}}_{2}^{\circ -},\cdots ,{\bar{g}}_{n}^{\circ -})$ *be the positive and negative ideal greyness degree vector respectively. Here the following notation is used.*

$${\hat{\bar{⊗}}}_{j}^{+}=\left\{ \begin{array}{l} \underset{i}{\max}\;y_{ij},\;j=1,2,\cdots ,k\\ \underset{i}{\max}\;{\hat{⊗}}_{ij},\;j=k+1,k+2,\cdots ,s\\ \underset{i}{\max}\;{\hat{⊗}}_{ij}^{\pm },\;j=s+1,s+2,\cdots ,n \end{array}\right.\;{\hat{\bar{⊗}}}_{j}^{-}=\left\{ \begin{array}{l} \underset{i}{\min}\;y_{ij},\;j=1,2,\cdots ,k\\ \underset{i}{\min}\;{\hat{⊗}}_{ij},\;j=k+1,k+2,\cdots ,s\\ \underset{i}{\min}\;{\hat{⊗}}_{ij}^{\pm },\;j=s+1,s+2,\cdots ,n \end{array}\right.$$

$${\bar{g}}_{j}^{\circ +}=\left\{ \begin{array}{l} \underset{i}{\min}\;0_{ij}=0,\;j=1,2,\cdots ,k\\ \underset{i}{\min}\;g_{ij}^{\circ }(⊗),\;j=k+1,k+2,\cdots ,s\\ \underset{i}{\min}\;g_{ij}^{\circ }({⊗}^{\pm }),\;j=s+1,s+2,\cdots ,n \end{array}\right.\;{\bar{g}}_{j}^{\circ -}=\left\{ \begin{array}{l} \underset{i}{\max}\;0_{ij}=0,\;j=1,2,\cdots ,k\\ \underset{i}{\max}\;g_{ij}^{\circ }(⊗),\;j=k+1,k+2,\cdots ,s\\ \underset{i}{\max\;}g_{ij}^{\circ }({⊗}^{\pm }),\;j=s+1,s+2,\cdots ,n \end{array}\right.$$

**Definition** **8.** *Assume that* $\forall {\hat{\bar{⊗}}}_{ij}\in {\hat{\bar{⊗}}}_{i}$*,* ${\hat{\bar{⊗}}}_{j}^{+}\in {\hat{\bar{⊗}}}^{+}$*,* ${\hat{\bar{⊗}}}_{j}^{-}\in {\hat{\bar{⊗}}}^{-}$*, then the kernel grey relational coefficient* $r({\hat{\bar{⊗}}}_{ij},{\hat{\bar{⊗}}}_{j}^{+(-)})$ *between the kernel vector of alternative* $S_{i}(i=1,2,\cdots ,m)$ *and the positive (negative) ideal kernel vector at attribute* $b_{j}(j=1,2,\cdots ,n)$ *is expressed as follows:*

(9) $$r({\hat{\bar{⊗}}}_{ij},{\hat{\bar{⊗}}}_{j}^{+(-)})=\frac{\underset{i}{\min}\;\underset{j}{\min}\left|{\hat{\bar{⊗}}}_{ij}-{\hat{\bar{⊗}}}_{j}^{+(-)}\right|+\xi \underset{i}{\max}\;\underset{j}{\max}\left|{\hat{\bar{⊗}}}_{ij}-{\hat{\bar{⊗}}}_{j}^{+(-)}\right|}{\left|{\hat{\bar{⊗}}}_{ij}-{\hat{\bar{⊗}}}_{j}^{+(-)}\right|+\xi \underset{i}{\max}\;\underset{j}{\max}\left|{\hat{\bar{⊗}}}_{ij}-{\hat{\bar{⊗}}}_{j}^{+(-)}\right|}$$

*where* ξ∈(0,1) *is a distinguished coefficient, its value is usually configured to 0.5.*

The kernel $\left|{\hat{\bar{⊗}}}_{ij}-{\hat{\bar{⊗}}}_{j}^{+(-)}\right|$ represents the absolute difference between the kernel vector of alternative $S_{i}$ and positive (negative) ideal kernel at attribute $b_{j}$. The positive ideal kernel vector corresponds to the superscript +, and the negative ideal kernel vector corresponds to the superscript −.

**Definition** **9.** *Assume that* $\forall {\bar{g}}_{ij}^{\circ }\in {\bar{g}}_{i}^{\circ }$*,* ${\bar{g}}_{j}^{\circ +}\in {\bar{g}}^{\circ +}$*,* ${\bar{g}}_{j}^{\circ -}\in {\bar{g}}^{\circ -}$*, then the greyness degree grey relational coefficient* $r({\bar{g}}_{ij}^{\circ },{\bar{g}}_{j}^{\circ +(-)})$ *between the greyness degree vector of alternative* $S_{i}(i=1,2,\cdots ,m)$ *and the positive (negative) ideal greyness degree vector at attribute* $b_{j}(j=1,2,\cdots ,n)$ *is expressed as follows:*

(10) $$r({\bar{g}}_{ij}^{\circ },{\bar{g}}_{j}^{\circ +(-)})=\frac{\underset{i}{\min}\;\underset{j}{\min}\left|{\bar{g}}_{ij}^{\circ }-{\bar{g}}_{j}^{\circ +(-)}\right|+\xi \underset{i}{\max}\;\underset{j}{\max}\left|{\bar{g}}_{ij}^{\circ }-{\bar{g}}_{j}^{\circ +(-)}\right|}{\left|{\bar{g}}_{ij}^{\circ }-{\bar{g}}_{j}^{\circ +(-)}\right|+\xi \underset{i}{\max}\;\underset{j}{\max}\left|{\bar{g}}_{ij}^{\circ }-{\bar{g}}_{j}^{\circ +(-)}\right|}$$

*where* ξ∈(0,1) *is a distinguished coefficient, its value is usually configured to 0.5.*

The greyness degree $\left|{\bar{g}}_{ij}^{\circ }-{\bar{g}}_{j}^{\circ +(-)}\right|$ represents the absolute difference between the greyness degree vector of alternative $S_{i}$ and positive (negative) ideal greyness degree at attribute $b_{j}$. The positive ideal greyness degree vector corresponds to the superscript +, and the negative ideal greyness degree vector corresponds to the superscript −.

Obviously, the kernel grey relational coefficient between the positive (negative) ideal kernel vector and itself at each attribute is equal to 1. Similarly, the greyness degree grey relational coefficient between the positive (negative) ideal greyness degree vector and itself at each attribute is also equal to 1.

**Definition** **10.** *Assume that* $\alpha^{*+(-)}$ *is an augmented matrix formed by positive (negative) ideal kernel vector and kernel vectors of all alternatives, then the weighted grey relational kernel matrix* $\alpha^{*+(-)}$ *that is about positive (negative) ideal kernel vector under the effect of weight vector* $\left(w_{1}^{+(-)},w_{2}^{+(-)},\cdots ,w_{n}^{+(-)}\right)$ *can be constructed as follows:*

(11) $$\alpha_{w}^{+(-)}=\left[\begin{array}{cccc} w_{1}^{+(-)} & w_{2}^{+(-)} & \cdots & w_{n}^{+(-)}\\ w_{1}r({\hat{\bar{⊗}}}_{11},{\hat{\bar{⊗}}}_{1}^{+(-)}) & w_{2}r({\hat{\bar{⊗}}}_{12},{\hat{\bar{⊗}}}_{2}^{+(-)}) & \cdots & w_{n}r({\hat{\bar{⊗}}}_{1n},{\hat{\bar{⊗}}}_{n}^{+(-)})\\ \vdots & \vdots & \ddots & \vdots \\ w_{1}r({\hat{\bar{⊗}}}_{m1},{\hat{\bar{⊗}}}_{1}^{+(-)}) & w_{2}r({\hat{\bar{⊗}}}_{m2},{\hat{\bar{⊗}}}_{2}^{+(-)}) & \cdots & w_{n}r({\hat{\bar{⊗}}}_{mn},{\hat{\bar{⊗}}}_{n}^{+(-)}) \end{array}\right]$$

*where the positive ideal kernel vector corresponds to the superscript* +*, and the negative ideal kernel vector corresponds to the superscript −.*

Similarly, the weighted grey relational greyness degree matrix can be constructed and denoted as $\beta_{w}^{+(-)}$, where the positive ideal greyness degree vector corresponds to the superscript +, and the negative ideal greyness degree vector corresponds to the superscript −.

**Definition** **11.** *Assume that* $\delta_{\alpha }^{+}(\delta_{\alpha }^{-})$ *is the kernel positive (negative) ideal alternative in* $\alpha_{w}^{+(-)}$*, where* $\delta_{\alpha }^{+}(\delta_{\alpha }^{-})=(w_{1}^{+(-)},w_{2}^{+(-)},\cdots ,w_{n}^{+(-)})=(w_{1},w_{2},\cdots ,w_{n})$*, and let the (i + 1)-th* (i=1,2,⋯,m) *row vector* $\alpha_{i}$ *be called the alternative $S_{i}$. Then the projection value* $Prj_{\delta_{\alpha }^{+}}\alpha_{i}$ *of alternative* $S_{i}$ *on the kernel positive ideal alternative* $\delta_{\alpha }^{+}$ *is expressed as follows:*

(12) $$Prj_{\delta_{\alpha }^{+}}\alpha_{i}=\frac{\alpha_{i}\cdot \delta_{\alpha }^{+}}{\left|\delta_{\alpha }^{+}\right|}=\frac{\sum_{j=1}^{n}w_{j}\cdot r({\hat{\bar{⊗}}}_{ij},{\hat{\bar{⊗}}}_{j}^{+})\cdot w_{j}}{\sqrt{\sum_{j=1}^{n}{\left(w_{j}\right)}^{2}}}=\sum_{j=1}^{n}r({\hat{\bar{⊗}}}_{ij},{\hat{\bar{⊗}}}_{j}^{+})\frac{w_{j}{}^{2}}{\sqrt{\sum_{j=1}^{n}{\left(w_{j}\right)}^{2}}}$$

Similarly, the projection value $Prj_{\delta_{\alpha }^{-}}\alpha_{i}$ of alternative $S_{i}$ on the kernel negative ideal alternative $\delta_{\alpha }^{-}$ is expressed as follows:

(13) $$Prj_{\delta_{\alpha }^{-}}\alpha_{i}=\frac{\alpha_{i}\cdot \delta_{\alpha }^{-}}{\left|\delta_{\alpha }^{-}\right|}=\frac{\sum_{j=1}^{n}w_{j}\cdot r({\hat{\bar{⊗}}}_{ij},{\hat{\bar{⊗}}}_{j}^{-})\cdot w_{j}}{\sqrt{\sum_{j=1}^{n}{\left(w_{j}\right)}^{2}}}=\sum_{j=1}^{n}r({\hat{\bar{⊗}}}_{ij},{\hat{\bar{⊗}}}_{j}^{-})\frac{w_{j}{}^{2}}{\sqrt{\sum_{j=1}^{n}{\left(w_{j}\right)}^{2}}}$$

The larger the $Prj_{\delta_{\alpha }^{+}}\alpha_{i}$ is, the closer the alternative $S_{i}$ is to the kernel positive ideal alternative, and the more consistent the changing direction between them will be. The smaller the $Prj_{\delta_{\alpha }^{-}}\alpha_{i}$ is, the further the alternative $S_{i}$ is from the kernel negative ideal alternative, and the more inconsistent the changing direction between them will be.

**Definition** **12.** *Assume that* $\delta_{\beta }^{+}(\delta_{\beta }^{-})$ *is the greyness degree positive (negative) ideal alternative in* $\beta_{w}^{+(-)}$*, where* $\delta_{\beta }^{+}(\delta_{\beta }^{-})=(w_{1}^{+(-)},w_{2}^{+(-)},\cdots ,w_{n}^{+(-)})=(w_{1},w_{2},\cdots ,w_{n})$*, and let the (i + 1)-th* (i=1,2,⋯,m) *row vector* $\beta_{i}$ *be called the alternative* $S_{i}$*. Then the projection value* $Prj_{\delta_{\beta }^{+}}\beta_{i}$ *of alternative* $S_{i}$ *on the greyness degree positive ideal alternative* $\delta_{\beta }^{+}$ *is expressed as follows:*

(14) $$Prj_{\delta_{\beta }^{+}}\beta_{i}=\frac{\beta_{i}\cdot \delta_{\beta }^{+}}{\left|\delta_{\beta }^{+}\right|}=\frac{\sum_{j=1}^{n}w_{j}\cdot r({\bar{g}}_{ij}^{\circ },{\bar{g}}_{j}^{\circ +})\cdot w_{j}}{\sqrt{\sum_{j=1}^{n}{\left(w_{j}\right)}^{2}}}=\sum_{j=1}^{n}r({\bar{g}}_{ij}^{\circ },{\bar{g}}_{j}^{\circ +})\frac{w_{j}{}^{2}}{\sqrt{\sum_{j=1}^{n}{\left(w_{j}\right)}^{2}}}$$

Similarly, the projection value $Prj_{\delta_{\beta }^{-}}\beta_{i}$ of alternative $S_{i}$ on the greyness degree negative ideal alternative $\delta_{\beta }^{-}$ is expressed as follows:

(15) $$Prj_{\delta_{\beta }^{-}}\beta_{i}=\frac{\beta_{i}\cdot \delta_{\beta }^{-}}{\left|\delta_{\beta }^{-}\right|}=\frac{\sum_{j=1}^{n}w_{j}\cdot r({\bar{g}}_{ij}^{\circ },{\bar{g}}_{j}^{\circ -})\cdot w_{j}}{\sqrt{\sum_{j=1}^{n}{\left(w_{j}\right)}^{2}}}=\sum_{j=1}^{n}r({\bar{g}}_{ij}^{\circ },{\bar{g}}_{j}^{\circ -})\frac{w_{j}{}^{2}}{\sqrt{\sum_{j=1}^{n}{\left(w_{j}\right)}^{2}}}$$

The larger the $Prj_{\delta_{\beta }^{+}}\beta_{i}$ is, the closer the alternative $S_{i}$ is to greyness degree positive ideal alternative, and the more consistent the changing direction between them will be. The smaller the $Prj_{\delta_{\beta }^{-}}\beta_{i}$ is, the further the alternative $S_{i}$ is from greyness degree negative ideal alternative, and the more inconsistent the changing direction between them will be.

In making a decision on alternatives, the closer the alternative $S_{i}$ is to the kernel positive ideal alternative and the further it is away from the kernel negative ideal alternative, the better the alternative $S_{i}$ is. However, it is difficult to meet the above requirements simultaneously in the actual decision-making process. In view of this, the following objective function is established according to the principle of minimum square summation:

(16) $$\min F(\phi_{i})={\left[\phi_{i}(Prj_{\delta_{\alpha }^{+}}\alpha_{i}-\left|\delta_{\alpha }^{+}\right|)\right]}^{2}+{\left[\left(1-\phi_{i})(Prj_{\delta_{\alpha }^{-}}\alpha_{i}-\left|\delta_{\alpha }^{-}\right|\right)\right]}^{2}$$

Let $\frac{\partial F(\phi_{i})}{\partial \phi_{i}}=0$, then the kernel optimal membership degree $\phi_{i}$ of alternative $S_{i}$ is obtained as follows:

(17) $$\phi_{i}=\frac{{\left(Prj_{\delta_{\alpha }^{-}}\alpha_{i}-\left|\delta_{\alpha }^{-}\right|\right)}^{2}}{{\left(Prj_{\delta_{\alpha }^{-}}\alpha_{i}-\left|\delta_{\alpha }^{-}\right|\right)}^{2}+{\left(Prj_{\delta_{\alpha }^{+}}\alpha_{i}-\left|\delta_{\alpha }^{+}\right|\right)}^{2}}$$

The larger the $\phi_{i}$ is, the closer the alternative $S_{i}$ is to kernel positive ideal alternative; the smaller the $\phi_{i}$ is, the further the alternative $S_{i}$ is from kernel negative ideal alternative. Similarly, the greyness degree optimal membership degree $\eta_{i}$ of alternative $S_{i}$ could be obtained and denoted as follows:

(18) $$\eta_{i}=\frac{{\left(Prj_{\delta_{\beta }^{-}}\beta_{i}-\left|\delta_{\beta }^{-}\right|\right)}^{2}}{{\left(Prj_{\delta_{\beta }^{-}}\beta_{i}-\left|\delta_{\beta }^{-}\right|\right)}^{2}+{\left(Prj_{\delta_{\beta }^{+}}\beta_{i}-\left|\delta_{\beta }^{+}\right|\right)}^{2}}$$

The larger the $\eta_{i}$ is, the closer the alternative $S_{i}$ is to greyness degree positive ideal alternative; the smaller the $\eta_{i}$ is, the further the alternative $S_{i}$ is from greyness degree negative ideal alternative.

**Definition** **13.** *Assume that* $\phi_{i}$ *and* $\eta_{i}$ *are kernel optimal membership degree and greyness degree optimal membership degree of alternative* $S_{i}$ *respectively, then comprehensive optimal membership degree* $G_{i}$ *of alternative* $S_{i}$ *is expressed as follows:*

(19) $$G_{i}=\phi_{i}+\eta_{i}$$

*The larger the* $G_{i}$ *is, the better the alternative* $S_{i}$ *is.*

### 4.3. Determination of Hierarchical Attribute Weights Based on HG-DEMATEL

In the decision-making problem where the decision information consists of grey multi-source heterogeneous data and the attribute system has a multi-hierarchical structure, there usually exist causalities between attributes. However, the determination of the traditional objective attribute weights mainly depends on attribute values distribution, and assumes that attributes are independent of each other. It can be seen that the causalities between attributes are ignored. The advantage of the DEMATEL method is that it can comprehensively use graph theory and matrix theory to analyze the causalities between complex system factors. It is especially more effective for the system with uncertain causalities between factors [47,48].

The initial direct relation matrix is the basis for causalities analysis of DEMATEL method, which has an important influence on final result of causalities analysis. It is usually determined by group multi-expert information aggregation. However, the current research mostly uses the mean method to aggregate the group information, which lacks scientific rationality. Therefore, in order to effectively aggregate experts’ evaluation information, the HG-DEMATEL method is proposed based on comprehensive consideration of differences and similarity degrees between initial direct relation matrixes given by experts. The proposed method is used to analyze the causalities between attributes and calculate the hierarchical attribute weights. The specific steps are as follows:

Step 1: Determine the direct relationships between attributes which form the attributes set $B=\left\{b_{1},b_{2},\cdots ,b_{n}\right\}$.

Step 2: Construct the overall initial direct relation matrix $Z={\left[z_{je}\right]}_{n\times n}$.

Assume that $Z^{\left(u\right)}={\left[z_{je}^{\left(u\right)}\right]}_{n\times n}$ is initial direct relation matrix constructed by the *u*-th expert based on an integer scale varying from 0, 1, 2, 3, 4, representing “no influence”, “very low influence”, “low influence”, “high influence”, and “very high influence” respectively. A total of q initial direct relation matrixes $Z^{\left(1\right)}$, $Z^{\left(2\right)}$, ⋯, $Z^{\left(q\right)}$ are aggregated into overall initial direct relation matrix $Z={\left[z_{je}\right]}_{n\times n}$ according to the following two objectives:

(1) The difference value between aggregated matrix and original matrix is as small as possible.
(2) The similarity degree value between aggregated matrix and original matrix is as big as possible.

Therefore, an optimization model $M_{1}$ is constructed based on the above two objectives which are the minimum difference and the similarity degree reached the critical value ξ.

(20) $$\begin{array}{l} M_{1}:\;\min T=\sum_{u=1}^{q}\left[\sqrt{\sum_{j=1}^{n}\sum_{e=1}^{n}{\left(z_{je}-z_{je}^{\left(u\right)}\right)}^{2}}\right]\\ \;s.t.\left\{ \begin{array}{l} R(Z^{\left(u\right)},Z)=\frac{<Z^{\left(u\right)},Z>}{\left\|Z^{\left(u\right)}\right\|\cdot \left\|Z\right\|}=\frac{<Z^{\left(u\right)},Z>}{\sqrt{<Z^{\left(u\right)},Z^{\left(u\right)}>}\cdot \sqrt{<Z,Z>}}\geq \xi ,\;u=1,2,\cdots ,q\\ z_{je}\in [0,4] \end{array}\right. \end{array}$$

where $R(Z^{\left(u\right)},Z)\in [0,1]$ is the similarity degree of the matrices $Z^{\left(u\right)}$ and Z, $<Z^{\left(u\right)},Z>$ is the inner product of the matrices $Z^{\left(u\right)}$ and Z. The inner product is the accumulative sum of the product of corresponding position element of two matrices [49]. The symbols $\left\|Z^{\left(u\right)}\right\|$ and ‖Z‖ stand for matrix norms; in fact they denote Hilbert-Schmidt norms. The model $M_{1}$ is solved according to the given critical value ξ and the overall initial direct relation matrix $Z={\left[z_{je}\right]}_{n\times n}$ is obtained.

Step 3: Construct the normalized direct relation matrix M, where M=γZ and $\gamma =1/\max(\sum_{e=1}^{n}z_{je})$.

Step 4: Calculate the total relation matrix T.

The total relation matrix is the sum of direct relation matrix and indirect relation matrix, where the indirect relation matrix consists of a decreasing matrix sequence of $M^{2},M^{3},\cdots ,M^{g}$, and $\underset{g\to \infty }{\lim}M^{g}={\left[0\right]}_{n\times n}$, that is:

(21) $$T=\underset{g\to \infty }{\lim}(M+M^{2}+\cdots +M^{g})=M{\left(I-M\right)}^{-1}$$

where I is unit matrix.

Step 5: Calculate the structural correlation coefficients.

The sum of the *j*-th row and sum of the *e*-th column in T are denoted as $R_{j}$ and $C_{e}$ respectively, so that:

(22) $$R_{j}=\sum_{e=1}^{n}t_{je}\;(j=1,2,\cdots ,n)$$

(23) $$C_{e}=\sum_{j=1}^{n}t_{je}\;(e=1,2,\cdots ,n)$$

In order to get a causal diagram, the values of $R_{j}+C_{j}$ and $R_{j}-C_{j}$ are calculated. The causal diagram can be obtained by mapping $\left(R_{j}+C_{j},R_{j}-C_{j}\right)$ in coordinates, where the horizontal axis $R_{j}+C_{j}$ is called “prominence” and the vertical axis $R_{j}-C_{j}$ is called “net effect”. In the causal diagram, the prominence axis shows the relative importance of each attribute in the attributes set, and the net effect axis divides the attributes set into cause and effect groups. When $R_{j}-C_{j}>0$, it means that the attribute $b_{j}$ belongs to the cause group and that it is a net causer; when $R_{j}-C_{j}<0$, it means that the attribute $b_{j}$ belongs to the effect group and that it is a net receiver. Hence, the complex causalities between attributes could be revealed by the structural correlation coefficients and visualized in the obtained causal diagram.

Step 6: Calculate the hierarchical attribute weights.

In this step, we use the structural correlation coefficients to set the attribute weights that will be used in the decision process. The weight $W_{j}^{\prime }$ of attribute $b_{j}$ is calculated as follows:

(24) $$W_{j}^{\prime }=\sqrt{{\left(R_{j}+C_{j}\right)}^{2}+{\left(R_{j}-C_{j}\right)}^{2}}$$

The weight $W_{j}^{\prime }$ of attribute $b_{j}$ can be normalized as follows:

(25) $$W_{j}=W_{j}^{\prime }/\sum_{j=1}^{n}W_{j}^{\prime }$$

So the normalized attribute weight vector $W_{MA}=(W_{1},W_{2},\cdots ,W_{n})$ can be obtained. Similarly, the sub-attribute weight vector $W_{SA}$ can also be calculated, and then the overall weight $w_{jf}$ of the *f*-th sub-attribute under the *j*-th attribute is defined as follows:

(26) $$w_{jf}=W_{j}W_{jf}$$

where $W_{j}$ is the weight of the *j*-th attribute in $W_{MA}$, and $W_{jf}$ is the weight of the *f*-th sub-attribute in $W_{SA}$.

### 4.4. The Decision-Making Steps

In summary, the main steps of the proposed decision-making method with grey multi-source heterogeneous data are given as follows:

Step 1: Normalize the comprehensive decision matrix with Equations (1)–(6);

Step 2: Construct the kernel vector and greyness degree vector of grey multi-source heterogeneous data sequence with Equations (7) and (8), then the positive and negative ideal kernel vector as well as the positive and negative ideal greyness degree vector are determined according to Definition 7;

Step 3: Calculate the hierarchical attribute weights with Equations (20)–(26);

Step 4: Calculate the projection values of each alternative on kernel positive and negative ideal alternative as well as greyness degree positive and negative ideal alternative with Equations (9)–15);

Step 5: Calculate the comprehensive optimal membership degree of each alternative with Equations (16)–(19);

Step 6: Rank alternatives. Sorting the values of the comprehensive optimal membership degree $G_{i}\;(i=1,2,\cdots ,m)$ in a descending sequence. The larger the value of $G_{i}$, the better the preference of the alternative $S_{i}$.

## 5. Case Study

In this section, a case of green supplier selection about product material component is presented based on the proposed method.

### 5.1. Case Background

With the increasingly serious environmental problems, the green supply chain management (GSCM) has been accepted as a modern management mode. Because green supplier is the upstream of the entire supply chain, its function in cost savings and environmental protection can pass through the supply chain to all downstream links [50]. It can bring a competitive advantage to the entire supply chain.

Hence, a decision problem of a product material component manufacturer is taken as an example, which aims to select the best green supplier for production. The four potential green suppliers are denoted as $S_{1}$, $S_{2}$, $S_{3}$, $S_{4}$, respectively. We analyze the literature which derives from the literature review of the green supplier selection in Section 2. Meanwhile, we consult some experts and perform practical investigation in some enterprises. Then a set of green supplier selection attributes are identified. The attributes including the product competitiveness, the enterprise competitiveness and the cooperation support are selected as the primary conventional attributes and the green level is selected as the green attribute. Similarly, we arrive at thirteen sub-attributes. The attributes of the green supplier selection are shown in Table 1.

In order to evaluate the potential green suppliers objectively and comprehensively, we investigate and obtain the decision information from multiple sources. Meanwhile, owing to the complexity and uncertainty of the actual decision environment, the obtained decision information is usually grey number, and then the fusion of different data types of grey information from multiple sources forms grey multi-source heterogeneous data sequence. The decision information about the four green suppliers are shown in Table 2, where the data of sub-attributes $b_{1}$ (%), $b_{2}$ (score), $b_{3}$ (score) are obtained from the procurement department and the material controlling department; the data of sub-attributes $b_{4}$ (%), $b_{5}$ (yuan), $b_{6}$ (%) are obtained from the production department and the quality management department; the data of sub-attributes $b_{7}$ (score), $b_{8}$ (score), $b_{9}$ (%), $b_{10}$ (score) are obtained from the financial department and the technology department; the data of sub-attributes $b_{11}$ (score), $b_{12}$ (score), $b_{13}$ (%) are obtained from the after-sale service department and the logistics department.

To select the best green supplier for production, the proposed method with grey multi-source heterogeneous data is used to solve the green supplier selection problem. The main rationale is that the kernel and greyness degree which form the common information characteristics of grey multi-source heterogeneous data are utilized to handle the decision information. Considering the multi-attribute multi-level decision structure in green supplier selection, the HG-DEMATEL method is used to capture the causalities between hierarchical attributes and determine the importance of hierarchical attributes. On this basis, the grey relational bi-directional projection method is used to rank the green suppliers, which can measure the proximity and the changing direction between the green suppliers and the positive (negative) ideal solution more accurately.

### 5.2. The Ranking of Alternatives

Step 1: Normalize the decision information in Table 2 with Equations (1)–(6), and the normalized comprehensive decision matrix is shown in Table 3.

Step 2: Construct the kernel vector and greyness degree vector of each alternative with Equations (7) and (8), then kernel matrix E organized by kernel vectors and greyness degree matrix F organized by greyness degree vectors are constructed, respectively.

$$E=\left[\begin{array}{ccccccccccccc} 0.40 & 0.25 & 0.40 & 0.30 & 0.67 & 0.14 & 0.31 & 0.32 & 0.40 & 0.34 & 0.32 & 0.18 & 0.56\\ 0.80 & 0.58 & 0.72 & 1.00 & 0.45 & 1.00 & 0.75 & 0.79 & 0.90 & 0.84 & 0.48 & 0.75 & 1.00\\ 1.00 & 0.37 & 0.42 & 0.70 & 0.56 & 0.53 & 0.36 & 0.65 & 1.00 & 0.41 & 0.73 & 0.39 & 0.00\\ 0.00 & 0.45 & 0.50 & 0.00 & 0.36 & 0.00 & 0.42 & 0.18 & 0.00 & 0.16 & 0.77 & 0.32 & 1.00 \end{array}\right]$$

$$F=\left[\begin{array}{ccccccccccccc} 0 & 0.18 & 0.28 & 0 & 0.22 & 0 & 0.61 & 0.41 & 0 & 0.31 & 0.21 & 0.36 & 0\\ 0 & 0.32 & 0.24 & 0 & 0.22 & 0 & 0.50 & 0.41 & 0 & 0.31 & 0.24 & 0.50 & 0\\ 0 & 0.26 & 0.24 & 0 & 0.22 & 0 & 0.39 & 0.24 & 0 & 0.31 & 0.17 & 0.36 & 0\\ 0 & 0.21 & 0.24 & 0 & 0.28 & 0 & 0.61 & 0.35 & 0 & 0.31 & 0.17 & 0.36 & 0 \end{array}\right]$$

Then, the positive and negative ideal kernel vector ${\hat{\bar{⊗}}}^{+}$, ${\hat{\bar{⊗}}}^{-}$ as well as the positive and negative ideal greyness degree vector ${\bar{g}}^{\circ +}$, ${\bar{g}}^{\circ -}$ are determined according to Definition 7.

$${\hat{\bar{⊗}}}^{+}=(1.00,0.58,0.72,1.00,0.67,1.00,0.75,0.79,1.00,0.84,0.77,0.75,1.00)$$

$${\hat{\bar{⊗}}}^{-}=(0.00,0.25,0.40,0.00,0.36,0.00,0.31,0.18,0.00,0.16,0.32,0.18,0.00)$$

$${\bar{g}}^{\circ +}=(0,0.18,0.24,0,0.22,0,0.39,0.24,0,0.31,0.17,0.36,0)$$

$${\bar{g}}^{\circ -}=(0,0.32,0.28,0,0.28,0,0.61,0.41,0,0.31,0.24,0.50,0)$$

Step 3: Calculate hierarchical attribute weights.

Firstly, the initial direct relation matrixes $Z^{\left(1\right)}$, $Z^{\left(2\right)}$, and $Z^{\left(3\right)}$ that are given by three experts based on an integer scale varying from 0, 1, 2, 3, 4 are shown in Table 4.

Secondly, the overall initial direct relation matrix $Z={\left[z_{je}\right]}_{4\times 4}$ aggregated by Equation (20) is calculated as follows:

$$Z={\left[z_{je}\right]}_{4\times 4}=\left[\begin{array}{cccc} 0 & 1.8381 & 1.3239 & 1.7361\\ 3.8381 & 0 & 0.8981 & 2.4258\\ 1.8381 & 1.5742 & 0 & 2.8980\\ 1.7361 & 0.1619 & 0.8381 & 0 \end{array}\right]$$

Then, the total relation matrix T is calculated by Equation (21);

$$T=\left[\begin{array}{cccc} 0.5665 & 0.5153 & 0.4399 & 0.7323\\ 1.1173 & 0.4172 & 0.4956 & 0.9514\\ 0.8518 & 0.5325 & 0.3326 & 0.9260\\ 0.5047 & 0.2193 & 0.2738 & 0.3074 \end{array}\right]$$

Finally, the weights of attributes are calculated by Equations (22)–(25) and shown in Table 5.

Similarly, the normalized weights and overall weights of sub-attributes can be calculated by Equations (20)–(26), and the results are shown in Table 6.

Step 4: The projection values of each alternative $S_{i}(i=1,2,3,4)$ on kernel positive ideal alternative $\delta_{\alpha }^{+}$ and kernel negative ideal alternative $\delta_{\alpha }^{-}$ are calculated as follows respectively:

$$Prj_{\delta_{\alpha }^{+}}\alpha_{1}=0.1607,\;Prj_{\delta_{\alpha }^{+}}\alpha_{2}=0.2553,\;Prj_{\delta_{\alpha }^{+}}\alpha_{3}=0.2020,\;Prj_{\delta_{\alpha }^{+}}\alpha_{4}=0.1694$$

$$Prj_{\delta_{\alpha }^{-}}\alpha_{1}=0.2226,\;Prj_{\delta_{\alpha }^{-}}\alpha_{2}=0.1498,\;Prj_{\delta_{\alpha }^{-}}\alpha_{3}=0.1874,\;Prj_{\delta_{\alpha }^{-}}\alpha_{4}=0.2415$$

Similarly, the projection values of each alternative $S_{i}(i=1,2,3,4)$ on greyness degree positive ideal alternative $\delta_{\beta }^{+}$ and greyness degree negative ideal alternative $\delta_{\beta }^{-}$ are calculated as follows respectively:

$$Prj_{\delta_{\beta }^{+}}\beta_{1}=0.2536,\;Prj_{\delta_{\beta }^{+}}\beta_{2}=0.2419,\;Prj_{\delta_{\beta }^{+}}\beta_{3}=0.2779,\;Prj_{\delta_{\beta }^{+}}\beta_{4}=0.2585$$

$$Prj_{\delta_{\beta }^{-}}\beta_{1}=0.2226,\;Prj_{\delta_{\beta }^{-}}\beta_{2}=0.1498,\;Prj_{\delta_{\beta }^{-}}\beta_{3}=0.1874,\;Prj_{\delta_{\beta }^{-}}\beta_{4}=0.2415$$

Step 5: Calculate the comprehensive optimal membership degree $G_{i}$ of each alternative $S_{i}$.

The kernel optimal membership degree $\phi_{i}$ and greyness degree optimal membership degree $\eta_{i}$ of each alternative $S_{i}(i=1,2,3,4)$ are calculated as follows respectively:

$$\phi_{1}=0.2033,\;\phi_{2}=0.9518,\;\phi_{3}=0.5797,\;\phi_{4}=0.1271$$

$$\eta_{1}=0.7935,\;\eta_{2}=0.9053,\;\eta_{3}=0.9934,\;\eta_{4}=0.7241$$

According to the kernel optimal membership degree $\phi_{i}$, the ranking result is $S_{2}≻S_{3}≻S_{1}≻S_{4}$, which has a deeper insight into the effect of potentially truly unknown values of grey number on the decision results. According to the greyness degree optimal membership degree $\eta_{i}$, the ranking result is $S_{3}≻S_{2}≻S_{1}≻S_{4}$, which takes the uncertain extent of grey number into account. Then, we consider the kernel optimal membership degree $\phi_{i}$ and greyness degree optimal membership degree $\eta_{i}$ comprehensively, and the comprehensive optimal membership degree $G_{i}$ of each alternative $S_{i}(i=1,2,3,4)$ is calculated as follows respectively:

$$G_{1}=0.9968,\;G_{2}=1.8571,\;G_{3}=1.5732,\;G_{4}=0.8512$$

Step 6: Rank alternatives.

All alternatives are ranked based on the value $G_{i}$ of each alternative $S_{i}(i=1,2,3,4)$. Sorting the values of $G_{i}(i=1,2,3,4)$ in a descending sequence, and final ranking of alternative strategies is obtained as: $S_{2}≻S_{3}≻S_{1}≻S_{4}$, that is, the green supplier $S_{2}$ is the best, and the business decision-makers can give priority to cooperating with the supplier $S_{2}$.

### 5.3. Discussion and Implications

The selection of green supplier is a key step in green supply chain management (GSCM). The proposed decision-making method integrating the HG-DEMATEL and the grey relational bi-directional projection method provides a useful, practical and valid selection tool, which can improve the quality of green supplier selection decisions. We adopt the different data types of grey information from multiple sources to describe the potential green supplier’s comprehensive performance objectively and comprehensively. It is obvious that the decision information from multiple sources can be useful in improving the accuracy of the green supplier selection decisions. Meanwhile, the kernel and greyness degree that are the common information characteristics of grey multi-source heterogeneous data are utilized to process the decision information and avoid the loss of information conversion in green supplier selection effectively.

In making decisions about green supplier selection, the proposed grey relational bi-directional projection method is applied to evaluate the green suppliers. During the evaluation procedure, the distance proximity and the consistency of changing direction between each alternative and the ideal solution are simultaneously taken into consideration, which can more accurately measure the proximity and the similarity between them. By using the proposed method to evaluate and select the potential green suppliers, it can provide a reference value for how to choose the best green supplier to increase the competitiveness and economic level of the company. Moreover, the feasibility and effectiveness of the proposed method are further verified, which also provides some practical and theoretical guidance value for the enterprises that are implementing or will implement green supply chain management.

## 6. Result Analysis

In this section, the result analysis about the case in Section 5 is performed, which includes the causalities analysis, sensitivity analysis and comparative analysis in three parts.

### 6.1. Causalities Analysis

**Figure 1:**
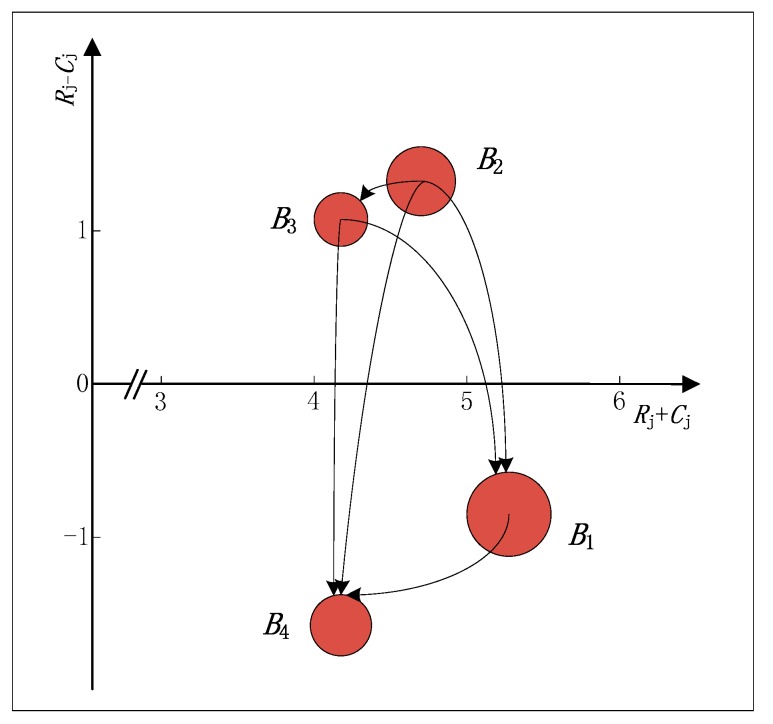
The causal diagram of attributes.

**Figure 2:**
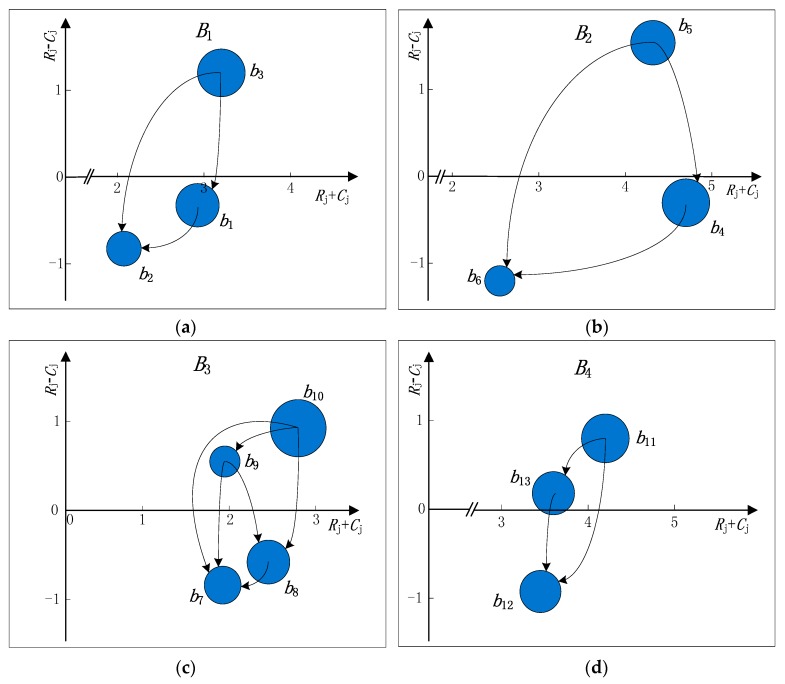
The causal diagram of sub-attributes. (**a**) The causal diagram of the sub-attributes contained in attribute *B*_1_; (**b**) The causal diagram of the sub-attributes contained in attribute *B*_2_; (**c**) The causal diagram of the sub-attributes contained in attribute *B*_3_; (**d**) The causal diagram of the sub-attributes contained in attribute *B*_4_.

The causal diagram of attributes (Figure 1) and the causal diagram of sub-attributes (Figure 2) can be made according to Table 5 and Table 6. In Figure 1 and Figure 2, the size of each bubble is relative to the normalized weight of each attribute or the overall weight of each sub-attribute. For example, the normalized weight of attribute $B_{2}$ is maximum, so it is represented by the biggest bubble. The causal diagram of attributes or sub-attributes provides the relative importance of each attribute or sub-attribute, and also analyzes the causalities between attributes or sub-attributes. The detailed analysis is as follows.

As can be seen from Table 5 and Figure 1, in terms of prominence, the prominence is at its highest at the green level $\left(B_{1}\right)$ and lowest at the enterprise competitiveness $\left(B_{3}\right)$ among the four evaluation attributes $B_{1},B_{2},B_{3},B_{4}$. It shows that the relative importance is strongest at $B_{1}$ and weakest at $B_{3}$ in the overall structure. In terms of net effect, the product competitiveness $\left(B_{2}\right)$ and the enterprise competitiveness $\left(B_{3}\right)$ have a positive net effect, and the green level $\left(B_{1}\right)$ and cooperation support $\left(B_{4}\right)$ possess a negative net effect. It means that $B_{2}$ and $B_{3}$ are net causers which belong to the cause group, while $B_{1}$ and $B_{4}$ are net receivers which belong to the effect group. The net effect is highest at $B_{2}$ and lowest at $B_{4}$. It indicates that $B_{4}$ is the most influenced by other attributes, meanwhile, $B_{2}$ is the key attribute of the whole evaluation system and it should be considered as the most desirable attribute to be improved.

For sub-attributes corresponding to each attribute, we can get the following analysis from Table 6 and Figure 2:

(1) With respect to the green level $\left(B_{1}\right)$, the prominence and net effect of energy consumption $\left(b_{3}\right)$ are both highest, indicating that it is not only the most important sub-attribute, but also the cause of effecting $B_{1}$. Only by reducing $b_{3}$ first it can well solve the ambient severities $\left(b_{2}\right)$ whose net effect is the lowest.
(2) With respect to the product competitiveness $\left(B_{2}\right)$, the prominence of product quality $\left(b_{4}\right)$ is highest, indicating that it is the most important sub-attribute in $B_{2}$. Meanwhile, product price $\left(b_{5}\right)$ possesses the highest net effect and does so as the only positive net causer, which can affect the product quality $\left(b_{4}\right)$ and product performance $\left(b_{6}\right)$. Therefore, we should give priority to the highest $b_{5}$ in net effect to improve the lowest $b_{6}$ in net effect.
(3) With respect to the enterprise competitiveness $\left(B_{3}\right)$, the prominence and net effect of management level $\left(b_{10}\right)$ are both highest, indicating that it is not only the most important sub-attribute, but also the cause of effecting $B_{3}$. By improving the management level $\left(b_{10}\right)$ first it can well enhance the technical level $\left(b_{8}\right)$, the staff quality $\left(b_{9}\right)$, and especially the financial situation $\left(b_{7}\right)$ whose prominence and net effect are both lowest.
(4) With respect to the cooperation support $\left(B_{4}\right)$, the prominence and net effect of after-sale service capabilities $\left(b_{11}\right)$ are both highest. Meanwhile, customer satisfaction $\left(b_{12}\right)$ possesses the lowest net effect and does as the only negative net receiver. It indicates that the improvement in after-sale service capabilities $\left(b_{11}\right)$ can effectively increase customer satisfaction $\left(b_{12}\right)$.

### 6.2. Sensitivity Analysis

In order to validate the robustness of the final recommendation based on the proposed method in green supplier selection, the following two aspects are considered in this section: the sensitivity analysis for variation of attribute weight; the sensitivity analysis for variation of attribute number.

#### 6.2.1. The Sensitivity Analysis for Variation of Attribute Weight

In order to investigate the influence of the change of attribute weights on the produced results, the sensitivity analysis of attribute weights is made in this part. A total of 13 sub-attributes weights constitutes the weight vector $\left(w_{1},w_{2},\cdots ,w_{13}\right)$. Firstly, a perturbation variable φ is given to the first weight $w_{1}$, and we have $w_{1}^{\prime }=\varphi w_{1}$. Then, the other attributes weights will vary from the obtained new weight value $w_{1}^{\prime }$, let $w_{j}^{\prime }=\psi w_{j}\;(j=2,3,\cdots ,13)$. The new weight vector should be normalized according to the following formula:

(27) $$w_{1}^{\prime }+\sum_{j=2}^{13}w_{j}^{\prime }=1\;\Rightarrow \;\varphi w_{1}+\psi \sum_{j=2}^{13}w_{j}=1$$

and we have:

(28) $$\psi =\left(1-\varphi w_{1}\right)/\left(1-w_{1}\right)$$

**Figure 3:**
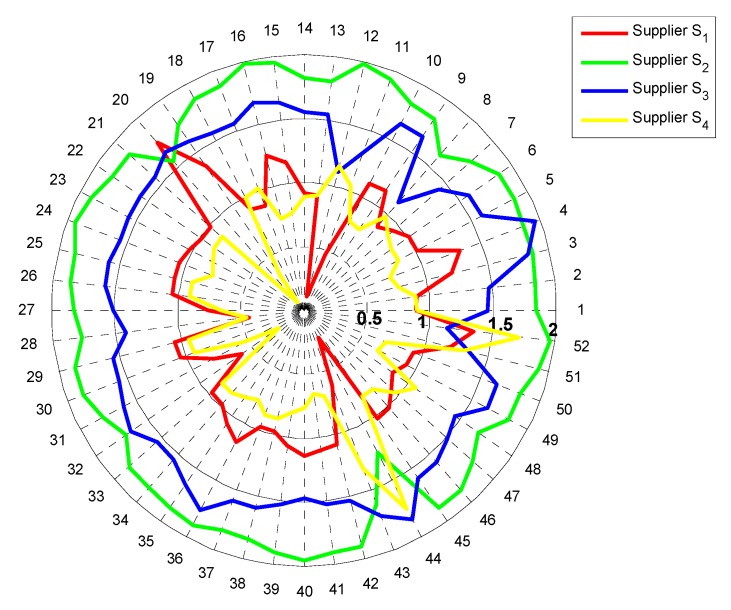
The result of the sensitivity analysis.

Hence, the new normalized weight vector $\left(w_{1}^{\prime },w_{2}^{\prime },\cdots ,w_{13}^{\prime }\right)$ is determined, and we set there are four scenarios when φ=1/3, 1/2, 2, 3 for the sensitivity analysis of $w_{1}$. Similarly, the same transformation is performed on the other 12 attributes, so a total of 52 scenarios can be obtained. The result of sensitivity analysis is shown in Figure 3.

From Figure 3, we can see that: (1) In total of 52 scenarios, supplier $S_{2}$ processes 48 times to be the optimal alternative, accounting for 92.3%, while supplier $S_{3}$ processes 47 times to be the suboptimal alternative, accounting for 90.4%. This indicates that the proposed decision-making method has lower sensitivity for the change of the ranking of the optimal alternative and suboptimal alternative, and the decision-making result is more reliable. (2) The supplier $S_{2}$ (the optimal alternative) is more sensitive than other potential suppliers for the disturbance of attributes $b_{1}$, $b_{5}$, $b_{11}$, while the supplier $S_{3}$ (the suboptimal alternative) is more sensitive to the disturbance of attributes $b_{1}$, $b_{3}$, $b_{11}$, $b_{13}$. In summary, the result of sensitivity analysis shows that the proposed decision-making method has high stability and reliability.

#### 6.2.2. The Sensitivity Analysis for Variation of Attribute Number

In order to further verify the validity of the final recommendation based on the proposed method for assessing green supplier, the sensitivity analysis for variation of attribute number is performed. In addition, in the green supplier selection process, the evaluation of alternatives may require the exclusion or addition of attributes. In this case, the proposed method should produce a robust and stable preference with the variation of attribute number.

**Figure 4:**
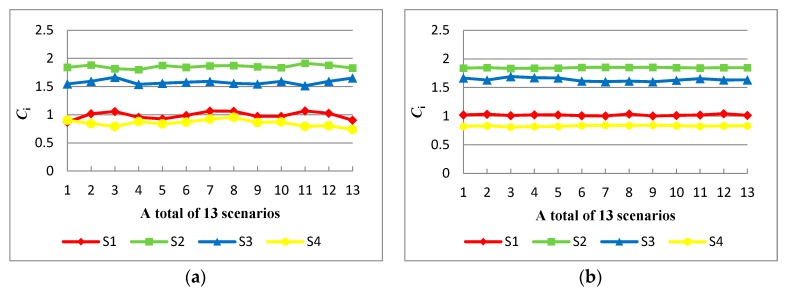
The sensitivity analysis for variation of attribute number. (**a**) The sensitivity analysis for the exclusion of attribute number; (**b**) The sensitivity analysis for the addition of attribute number.

In the original case, the ranking result of four alternatives with thirteen attributes is $S_{2}≻S_{3}≻S_{1}≻S_{4}$. To test the robustness of the final recommendation, thirteen scenarios were performed, each scenario with the exclusion of one of the thirteen existing attribute. After normalizing the attribute weights in 13 scenarios, the specific results are shown in Figure 4a. From Figure 4a, there is only one scenario that the ranking result is inconsistent with the result in the original case, accounting for 7.6%. Meanwhile, the alternative $S_{2}$ is always the optimal recommendation. Hence, the results of sensitivity analysis that one of the attributes removes each time have shown no significant changes in the alternative ranking.

Furthermore, the attribute green image $b_{14}$ (%) is added, which is represented by the ratio of green customers to total customers [40,44]. Thirteen scenarios are also performed, in which the attribute weight of $b_{14}$ varies from equaling the first attribute weight to equaling the last attribute weight orderly. After normalizing the attribute weights in 13 scenarios, the specific results are shown in Figure 4b. It is distinct that the ranking results in Figure 4b remain the same with all the scenarios.

From the above analysis, it is shown that the final recommendation $S_{2}≻S_{3}≻S_{1}≻S_{4}$ is robust and valid when we perform the exclusion or addition of attributes.

### 6.3. Comparative Analysis

In order to verify the rationality and effectiveness of the proposed method, we compare the proposed method with several related methods. The comparison results can be seen in Table 7, and the particularized discussions and analysis are depicted in the following.

(1) If we use the method in reference [51] to deal with the same problem in this paper, the value of relative kernel of each alternative $S_{i}(i=1,2,3,4)$ can be calculated respectively: $\delta ({⊗}_{1})=0.2067$, $\delta ({⊗}_{2})=0.5068$, $\delta ({⊗}_{3})=0.3969$, $\delta ({⊗}_{4})=0.2265$, then we have $\delta ({⊗}_{2})>\delta ({⊗}_{3})>\delta ({⊗}_{4})>\delta ({⊗}_{1})\Rightarrow$ $S_{2}≻S_{3}≻S_{4}≻S_{1}$.
(2) If we use the method in reference [52] to deal with the same problem in this paper, the value of comprehensive correlation degree of each alternative $S_{i}(i=1,2,3,4)$ can be obtained respectively:$\lambda_{1}=0.4661$, $\lambda_{2}=0.6282$, $\lambda_{3}=0.5616$, $\lambda_{4}=0.4783$, then we have $\lambda_{2}>\lambda_{3}>\lambda_{4}>\lambda_{1}\Rightarrow$ $S_{2}≻S_{3}≻S_{4}≻S_{1}$.
(3) If we use the classical GRA method to deal with the same problem in this paper, the value of grey relation degree of each alternative $S_{i}(i=1,2,3,4)$ can be obtained respectively: $\gamma_{1}=1.350$, $\gamma_{2}=1.413$, $\gamma_{3}=1.429$, $\gamma_{4}=1.328$, then we have $\gamma_{3}>\gamma_{2}>\gamma_{1}>\gamma_{4}\Rightarrow$ $S_{3}≻S_{2}≻S_{1}≻S_{4}$.
(4) If we use the classical TOPSIS method to deal with the same problem in this paper, the value of relative closeness of each alternative $S_{i}(i=1,2,3,4)$ can be obtained respectively: ${RC}_{1}=0.194$, ${RC}_{2}=0.294$, ${RC}_{3}=0.345$, ${RC}_{4}=0.167$, then we have ${RC}_{3}>{RC}_{2}>{RC}_{1}>{RC}_{4}\Rightarrow$ $S_{3}≻S_{2}≻S_{1}≻S_{4}$.

From Table 7, we can see that the best alternative is identical in the references [51,52] and our method. The reasons for the differences between references [51,52] with our method may be the determination thought of attribute weights. Our method is based on the proposed HG-DEMATE, which has no restrictions on the relationships between attributes. The methods in references [51,52] are based on the linear programming model, which need to assume that attributes are independent. In addition, the ranking results in classical GRA method and classical TOPSIS method are different from the proposed method. The reason for the distinction may be that the classical GRA method only considers the distance proximity between the alternatives and the ideal solution, while the proposed method not only measures the distance proximity between each alternative and the ideal solution, but also considers the consistency of changing direction between them. Moreover, in the classical TOPSIS method, the consistency of changing direction between each alternative and positive (negative) ideal solution is not involved. From the analysis above, the advantages of the proposed method can be summarized as follows:

(1) A common method of solving hierarchical attribute weights, in which the causal relationships exist between attributes, is under consideration. The determination of the traditional objective attribute weights mainly depends on attribute values distribution, which assumes that attributes are independent. However, there usually exist causal relationships between attributes in the multi-hierarchical attribute system. The proposed HG-DEMATEL method may be a suitable way to solve this problem.
(2) Different from the classical methods, such as TOPSIS, GRA, the proposed grey relational bi-directional projection method not only considers the measurement of distance proximity between alternatives and the ideal solution, but also takes the consistency of changing direction between them into account.
(3) The decision-making information is usually the single homogeneous data in classical decision-making method, such as VIKOR, GRA, TOPSIS, while the proposed method can handle the composite decision-making information with two or more different types of grey information from multiple sources, which is more in line with the presentation of practical decision information.

## 7. Conclusions

This paper studies the multi-attribute decision-making problem that the attribute values are grey multi-source heterogeneous data. We define the grey multi-source heterogeneous data sequence, and then the grey relational bi-directional projection ranking method is proposed based on kernel vector and greyness degree vector of the sequence. Meanwhile, the HG-DEMATEL method is proposed to solve the problem of causalities between attributes and determine the hierarchical attribute weights. Finally, an illustrative example verifies the validity of the proposed method in green supplier selection. At the same time, the proposed method also provides a perspective to solve the uncertain decision-making problem whose decision-making information is grey multi-source heterogeneous data.

In future research, we can further integrate DEMATEL and the Choquet integral to deal with the causalities between different sources attributes, and use fuzzy integral and fuzzy measure to fuse multi-source heterogeneous data. In addition, the proposed method can be further investigated to make it suitable for other similar supplier selection problem, such as low carbon supplier selection, strategic supplier selection and sustainable supplier selection.

## Figures and Tables

**Table 1:** The attributes of the green supplier selection.

Attribute	Sub-Attribute	Relevant Reference	Explanation
Green level (*B*_1_)	Resources recovery (*b*_1_)	[33,40,41]	The ratio of recovery to input
	Ambient severities (*b*_2_)	[32,40]	The severity of affecting environment
	Energy consumption (*b*_3_)	[32,33,40]	The extent of energy consumption
Product competitiveness (*B*_2_)	Product quality (*b*_4_)	[34,36,38]	The product qualified rate
	Product price (*b*_5_)	[33,34,44]	The price of purchased product itself
	Product performance (*b*_6_)	[34,38]	The failure rate of the qualified product
Enterprise competitiveness (*B*_3_)	Financial situation (*b*_7_)	[32,44]	The ability of fund raising and application
	Technical level (*b*_8_)	[30,33,38]	The level of technical knowledge owned by an enterprise
	Staff quality (*b*_9_)	[38,39]	The proportion of middle or senior title within an enterprise
	Management level (*b*_10_)	[32,38,40]	The comprehensive management ability of an enterprise in the whole operation process
Cooperation support (*B*_4_)	After-sale service capabilities (*b*_11_)	[38,41]	The capabilities of various services provided after the sale of a product
	Customer satisfaction (*b*_12_)	[39,44]	The degree of satisfaction with products and related services
	Delivery on time (*b*_13_)	[33,36,38]	The ratio of the number of punctual deliveries to the number of total deliveries in a certain period of time

**Table 2:** Decision information.

		Potential Green Suppliers			
		*S*_1_	*S*_2_	*S*_3_	*S*_4_
*B*_1_	*b*_1_	33	35	36	31
	*b*_2_	[0.21,0.25]∪[0.28,0.31]	[0.12,0.20]∪[0.22,0.26]	[0.18,0.23]∪[0.25,0.30]	[0.17,0.21]∪[0.24,0.28]
	*b*_3_	[0.28,0.32]∪[0.35,0.45]	[0.20,0.25]∪[0.28,0.35]	[0.27,0.34]∪[0.36,0.41]	[0.26,0.31]∪[0.33,0.40]
*B*_2_	*b*_4_	91	98	95	88
	*b*_5_	[21,23]∪[25,27]	[23,25]∪[27,29]	[22,24]∪[26,28]	[23,26]∪[28,30]
	*b*_6_	8.8	5.7	7.4	9.3
*B*_3_	*b*_7_	[0.72,0.83]	[0.81,0.90]	[0.75,0.82]	[0.74,0.85]
	*b*_8_	[0.77,0.84]	[0.85,0.92]	[0.84,0.88]	[0.75,0.81]
	*b*_9_	27	32	33	23
	*b*_10_	[0.81,0.86]	[0.89,0.94]	[0.82,0.87]	[0.78,0.83]
*B*_4_	*b*_11_	[0.61,0.67]∪[0.70,0.73]	[0.65,0.70]∪[0.72,0.77]	[0.71,0.75]∪[0.78,0.81]	[0.73,0.76]∪[0.78,0.82]
	*b*_12_	[0.80,0.85]	[0.87,0.94]	[0.83,0.88]	[0.82,0.87]
	*b*_13_	91	95	86	95

**Table 3:** Normalized comprehensive decision matrix of decision information.

		Potential Green Suppliers			
		*S*_1_	*S*_2_	*S*_3_	*S*_4_
*B*_1_	*b*_1_	0.4	0.8	1	0
	*b*_2_	[0.00,0.16]∪[0.32,0.53]	[0.26,0.47]∪[0.58,1.00]	[0.05,0.32]∪[0.42,0.68]	[0.16,0.37]∪[0.53,0.74]
	*b*_3_	[0.00,0.40]∪[0.52,0.68]	[0.40,0.68]∪[0.80,1.00]	[0.16,0.36]∪[0.44,0.72]	[0.20,0.48]∪[0.56,0.76]
*B*_2_	*b*_4_	0.30	1.00	0.70	0.00
	*b*_5_	[0.33,0.56]∪[0.78,1.00]	[0.11,0.33]∪[0.56,0.78]	[0.22,0.44]∪[0.67,0.89]	[0.00,0.22]∪[0.44,0.78]
	*b*_6_	0.14	1.00	0.53	0.00
*B*_3_	*b*_7_	[0.00,0.61]	[0.50,1.00]	[0.17,0.56]	[0.11,0.72]
	*b*_8_	[0.12,0.53]	[0.59,1.00]	[0.53,0.76]	[0.00,0.35]
	*b*_9_	0.40	0.90	1.00	0.00
	*b*_10_	[0.19,0.50]	[0.69,1.00]	[0.25,0.56]	[0.00,0.31]
*B*_4_	*b*_11_	[0.00,0.29]∪[0.43,0.57]	[0.19,0.43]∪[0.52,0.76]	[0.48,0.67]∪[0.81,0.95]	[0.57,0.71]∪[0.81,1.00]
	*b*_12_	[0.00,0.36]	[0.50,1.00]	[0.21,0.57]	[0.14,0.50]
	*b*_13_	0.56	1.00	0.00	1.00

**Table 4:** Initial direct relation matrixes given by three experts.

	DM1				DM2				DM3			
	Initial Direct Relation Matrix $Z^{\left(1\right)}$				Initial Direct Relation Matrix $Z^{\left(2\right)}$				Initial Direct Relation Matrix $Z^{\left(3\right)}$			
	*B*_1_	*B*_2_	*B*_3_	*B*_4_	*B*_1_	*B*_2_	*B*_3_	*B*_4_	*B*_1_	*B*_2_	*B*_3_	*B*_4_
*B*_1_	0	2	1	1	0	2	1	2	0	1	3	2
*B*_2_	4	0	0	3	4	0	1	2	3	0	2	3
*B*_3_	2	1	0	2	2	2	0	3	1	1	0	4
*B*_4_	1	0	1	0	2	0	1	0	2	1	0	0

**Table 5:** Resulting weights of attributes.

Attributes	*R* + *C*	*R* − *C*	Weights	Normalized Weights
*B*_1_	5.2942	−0.7863	5.3523	0.2811
*B*_2_	4.6655	1.2972	4.8425	0.2543
*B*_3_	4.1847	1.1011	4.3272	0.2273
*B*_4_	4.2221	−1.6120	4.5193	0.2373

**Table 6:** Resulting weights of sub-attributes.

Sub-Attributes	*R* + *C*	*R* − *C*	Normalized Sub-Attribute Weights	Overall Sub-Attribute Weights
*b*_1_	2.8913	−0.3261	0.3422	0.0962
*b*_2_	2.0761	−0.8152	0.2623	0.0737
*b*_3_	3.1630	1.1413	0.3955	0.1112
*b*_4_	4.7123	−0.2740	0.3906	0.0993
*b*_5_	4.3014	1.5068	0.3772	0.0959
*b*_6_	2.5205	−1.2329	0.2322	0.0591
*b*_7_	1.9130	−0.8566	0.2208	0.0502
*b*_8_	2.4013	−0.5940	0.2606	0.0592
*b*_9_	1.9307	0.5217	0.2107	0.0479
*b*_10_	2.7714	0.9289	0.3079	0.0700
*b*_11_	4.2338	0.7792	0.3755	0.0891
*b*_12_	3.4545	−0.9091	0.3116	0.0739
*b*_13_	3.5844	0.1299	0.3129	0.0743

**Table 7:** The comparison results of several related methods.

Methods	Ranking Orders	Optimal Alternatives	Worst Alternatives
The method in reference [51]	$S_{2}≻S_{3}≻S_{4}≻S_{1}$	$S_{2}$	$S_{1}$
The method in reference [52]	$S_{2}≻S_{3}≻S_{4}≻S_{1}$	$S_{2}$	$S_{1}$
The classical GRA method	$S_{3}≻S_{2}≻S_{1}≻S_{4}$	$S_{3}$	$S_{4}$
The classical TOPSIS method	$S_{3}≻S_{2}≻S_{1}≻S_{4}$	$S_{3}$	$S_{4}$
The proposed method	$S_{2}≻S_{3}≻S_{1}≻S_{4}$	$S_{2}$	$S_{4}$

## References

[1] Jamshidi-Zanjani A., Rezaei M. (2017). Landfill site selection using combination of fuzzy logic and multi-attribute decision-making approach. Environ. Earth Sci..

[2] Kou G., Peng Y., Wang G.X. (2014). Evaluation of clustering algorithms for financial risk analysis using MCDM methods. Inf. Sci..

[3] Yin K.D., Wang P.Y., Li X.M. (2017). The Multi-Attribute Group Decision-Making Method Based on Interval Grey Trapezoid Fuzzy Linguistic Variables. Int. J. Environ. Res. Public Health.

[4] Chuu S.J. (2014). An investment evaluation of supply chain RFID technologies: A group decision-making model with multiple information sources. Knowl. Based Syst..

[5] Kou G., Ergu D., Lin C.S., Chen Y. (2016). Pairwise comparison matrix in multiple criteria decision making. Technol. Econ. Dev. Econ..

[6] Kou G., Lu Y.Q., Peng Y., Shi Y. (2012). Evaluation of classification algorithms using MCDM and rank correlation. Int. J. Inf. Technol. Decis. Mak..

[7] Wu W.S., Kou G. (2016). A group consensus model for evaluating real estate investment alternatives. Financ. Innov..

[8] Liu B.S., Yang X.D., Huo T.F., Shen G.Q., Wang X. (2017). A linguistic group decision-making framework for bid evaluation in mega public projects considering carbon dioxide emissions reduction. J. Clean. Prod..

[9] Yan S.L., Liu S.F., Liu J.F., Wu L.F. (2015). Dynamic grey target decision making method with grey numbers based on existing state and future development trend of alternatives. J. Intell. Fuzzy Syst..

[10] Hu M.L. (2016). Grey target decision model based on a new distance measure. J. Grey Syst..

[11] Zhou H., Wang J.Q., Zhang H.Y. (2017). Grey stochastic multi-criteria decision-making based on regret theory and TOPSIS. Int. J. Mach. Learn. Cybern..

[12] Lidinska L., Jablonsky J. (2017). AHP model for performance evaluation of employees in a Czech management consulting company. Cent. Eur. J. Oper. Res..

[13] Ji P., Zhang H.Y., Wang J.Q. (2017). A projection-based TODIM method under multi-valued neutrosophic environments and its application in personnel selection. Neural Comput. Appl..

[14] Hashemi S.S., Hajiagha S.H.R., Zavadskas E.K., Mahdiraji H.A. (2016). Multicriteria group decision making with ELECTRE III method based on interval-valued intuitionistic fuzzy information. Appl. Math. Model..

[15] Huang Y.B., Jiang W. (2017). Extension of TOPSIS Method and its Application in Investment. Arab. J. Sci. Eng..

[16] Zhou W., Yin W.Y., Peng X.Q., Liu F.M., Yang F. (2016). Comprehensive evaluation of land reclamation and utilisation schemes based on a modified VIKOR method for surface mines. Int. J. Min. Reclam. Environ..

[17] Deng J.L. (1989). Introduction to grey system theory. J. Grey Syst..

[18] Kolhapure R., Shinde V., Kamble V. (2017). Geometrical optimization of strain gauge force transducer using GRA method. Measurement.

[19] Kirubakaran B., Ilangkumaran M. (2016). Selection of optimum maintenance strategy based on FAHP integrated with GRA–TOPSIS. Ann. Oper. Res..

[20] Li N.N., Zhao H.R. (2016). Performance evaluation of eco-industrial thermal power plants by using fuzzy GRA-VIKOR and combination weighting techniques. J. Clean. Prod..

[21] Liu J., Guo L., Jiang J.P., Hao L.L., Liu R.T., Wang P. (2015). Evaluation and selection of emergency treatment technology based on dynamic fuzzy GRA method for chemical contingency spills. J. Hazard. Mater..

[22] Luo D., Wei B.L., Lin P.Y. (2015). The Optimization of Several Grey Incidence Analysis Models. J. Grey Syst..

[23] Wang P., Zhu Z.Q., Wang Y.H. (2016). A novel hybrid MCDM model combining the SAW, TOPSIS and GRA methods based on experimental design. Inf. Sci..

[24] Tsaur R.C., Chen I.F., Chan Y.S. (2017). TFT-LCD industry performance analysis and evaluation using GRA and DEA models. Int. J. Prod. Res..

[25] Ho T.C., Chiu R.H., Chung C.C., Lee H.S. (2017). Key influence factors for ocean freight forwarders selecting container shipping lines using the revised dematel approach. J. Mar. Sci. Technol..

[26] Hsu C.W., Kuo T.C., Chen S.H., Hu A.H. (2013). Using DEMATEL to develop a carbon management model of supplier selection in green supply chain management. J. Clean. Prod..

[27] Su C.M., Horng D.J., Tseng M.L., Chiu A.S., Wu K.J., Chen H.P. (2016). Improving sustainable supply chain management using a novel hierarchical grey-DEMATEL approach. J. Clean. Prod..

[28] Luthra S., Govindan K., Mangla S.K. (2017). Structural model for sustainable consumption and production adoption—A grey-DEMATEL based approach. Resour. Conserv. Recycl..

[29] Seker S., Recal F., Basligil H. (2017). A combined DEMATEL and grey system theory approach for analyzing occupational risks: A case study in Turkish shipbuilding industry. Hum. Ecol. Risk Assess..

[30] Govindan K., Kaliyan M., Kannan D., Haq A.N. (2014). Barriers analysis for green supply chain management implementation in Indian industries using analytic hierarchy process. Int. J. Prod. Econ..

[31] Yu Q., Hou F.J. (2016). An approach for green supplier selection in the automobile manufacturing industry. Kybernetes.

[32] Lin C.H., Madu C.N., Kuei C.H., Tsai H.L., Wang K.N. (2015). Developing an assessment framework for managing sustainability programs. Expert Syst. Appl..

[33] Govindan K., Kadziński M., Sivakumar R. (2017). Application of a novel PROMETHEE-based method for construction of a group compromise ranking to prioritization of green suppliers in food supply chain. Omega.

[34] Roshandel J., Miri-Nargesi S.S., Hatami-Shirkouhi L. (2013). Evaluating and selecting the supplier in detergent production industry using hierarchical fuzzy TOPSIS. Appl. Math. Model..

[35] Azadi M., Jafarian M., Saen R.F., Mirhedayatian S.M. (2015). A new fuzzy DEA model for evaluation of efficiency and effectiveness of suppliers in sustainable supply chain management context. Comput. Oper. Res..

[36] Teresa W., Jennifer B. (2009). Supplier evaluation and selection: An augmented DEA approach. Int. J. Prod. Res..

[37] Ghayebloo S., Tarokh M.J., Venkatadri U., Diallo C. (2015). Developing a bi-objective model of the closed-loop supply chain network with green supplier selection and disassembly of products: The impact of parts reliability and product greenness on the recovery network. J. Manuf. Syst..

[38] Amin S.H., Zhang G.Q. (2012). An integrated model for closed-loop supply chain configuration and supplier selection: Multi-objective approach. Expert Syst. Appl..

[39] Stanujkic D., Dordjevic B., Dordjevic M. (2013). Comparative analysis of some prominent MCDM methods: A case of ranking Serbian banks. Serb. J. Manag..

[40] Qin J.D., Liu X.W., Pedrycz W. (2017). An extended TODIM multi-criteria group decision making method for green supplier selection in interval type-2 fuzzy environment. Eur. J. Oper. Res..

[41] Tsui C.W., Wen U.P. (2014). A Hybrid Multiple Criteria Group Decision-Making Approach for Green Supplier Selection in the TFT-LCD Industry. Math. Probl. Eng..

[42] Hamdan S., Cheaitou A. (2017). Supplier selection and order allocation with green criteria: An MCDM and multi-objective optimization approach. Comput. Oper. Res..

[43] Bai C.G., Dhavale D., Sarkis J. (2016). Complex investment decisions using rough set and fuzzy c-means: An example of investment in green supply chains. Eur. J. Oper. Res..

[44] Luthra S., Govindan K., Kannan D., Mangla S.K., Garg C.P. (2017). An integrated framework for sustainable supplier selection and evaluation in supply chains. J. Clean. Prod..

[45] Liu S.F., Tao L.Y., Xie N.M., Yang Y.J. (2016). On the new model system and framework of grey system theory. J. Grey Syst..

[46] Yang Y.J. (2007). Extended grey numbers and their operations. Proceedings of the 2007 IEEE International Conference on Fuzzy Systems and Intelligent Services, Man and Cybernetics.

[47] Wu W.W., Lee Y.T. (2007). Developing global managers’ competencies using the fuzzy DEMATEL method. Expert Syst. Appl..

[48] Altuntas S., Dereli T. (2015). A novel approach based on DEMATEL method and patent citation analysis for prioritizing a portfolio of investment projects. Expert Syst. Appl..

[49] Nie W.B., Liu W.D., Wang J.D., Zeng T., Hu K. (2016). Evaluation approach in process failure risk analysis based on matrix similarity and evidence theory. Comput. Integr. Manuf. Syst..

[50] Kim M., Chai S. (2017). Implementing Environmental Practices for Accomplishing Sustainable Green Supply Chain Management. Sustainability.

[51] Guo S.D., Liu S.F., Fang Z.G. (2016). Multi-attribute decision making model based on kernel and degree of greyness of interval grey numbers. Control Decision.

[52] Liu Z.X., Liu S.F., Fang Z.G. (2017). Decision making model of grey comprehensive correlation and relative close degree based on kernel and greyness degree. Control Decision.

